# MIX-TPI: a flexible prediction framework for TCR–pMHC interactions based on multimodal representations

**DOI:** 10.1093/bioinformatics/btad475

**Published:** 2023-08-01

**Authors:** Minghao Yang, Zhi-An Huang, Wei Zhou, Junkai Ji, Jun Zhang, Shan He, Zexuan Zhu

**Affiliations:** College of Computer Science and Software Engineering, Shenzhen University, Shenzhen 518060, China; Research Office, City University of Hong Kong (Dongguan), Dongguan 523000, China; College of Computer Science and Software Engineering, Shenzhen University, Shenzhen 518060, China; College of Computer Science and Software Engineering, Shenzhen University, Shenzhen 518060, China; College of Computer Science and Software Engineering, Shenzhen University, Shenzhen 518060, China; School of Computer Science, University of Birmingham, Birmingham B15 2TT, United Kingdom; College of Computer Science and Software Engineering, Shenzhen University, Shenzhen 518060, China; National Engineering Laboratory for Big Data System Computing Technology, Shenzhen University, Shenzhen 518060, China

## Abstract

**Motivation:**

The interactions between T-cell receptors (TCR) and peptide-major histocompatibility complex (pMHC) are essential for the adaptive immune system. However, identifying these interactions can be challenging due to the limited availability of experimental data, sequence data heterogeneity, and high experimental validation costs.

**Results:**

To address this issue, we develop a novel computational framework, named MIX-TPI, to predict TCR–pMHC interactions using amino acid sequences and physicochemical properties. Based on convolutional neural networks, MIX-TPI incorporates sequence-based and physicochemical-based extractors to refine the representations of TCR–pMHC interactions. Each modality is projected into modality-invariant and modality-specific representations to capture the uniformity and diversities between different features. A self-attention fusion layer is then adopted to form the classification module. Experimental results demonstrate the effectiveness of MIX-TPI in comparison with other state-of-the-art methods. MIX-TPI also shows good generalization capability on mutual exclusive evaluation datasets and a paired TCR dataset.

**Availability and implementation:**

The source code of MIX-TPI and the test data are available at: https://github.com/Wolverinerine/MIX-TPI.

## 1 Introduction

T-cells play an essential part in regulating effector immune cells involved in the adaptive immune response against infections and cancer (Hudson and Wieland 2023). On the surface of T-cells, the T-cell receptor (TCR) is responsible for antigen recognition. Antigens are degraded into polypeptides, and antigenic peptides are bound to specific major histocompatibility complex (MHC) molecules in a process called peptide-MHC binding. TCR is a heterodimeric molecule consisting of two chains, i.e. an α-chain and a β-chain, which interact with peptide-MHC (pMHC) via six loops, i.e. three from the α-chain and three from β-chain. The three loops in each chain are known as complementarity determining regions (CDRs) 1–2–3. They are responsible for determining the TCR specificity. The CDR3 loops primarily interact with the peptide, while the CDR1 and CDR2 loops interact with MHC (Rossjohn *et al.* 2015). Upon recognition of pMHC by TCR, T-cells are stimulated to mount an immune response like proliferation, activation, or differentiation (Zhang *et al.* 2016). The characterization of TCR–pMHC interactions provides valuable insights into the development of personalized immunotherapies, including vaccine design and T-cell transfer methods (Frank *et al.* 2023). For instance, understanding the diversity of TCR and antibody repertoires can help reveal the adaptive immune status and history of patients. A number of wet lab experiments, such as tetramer analysis (Altman *et al.* 2011), tetramer-associated TCR sequencing (Zhang *et al.* 2018), and T-scans (Kula *et al.* 2019), have been conducted to investigate TCR–pMHC interactions. Nevertheless, these conventional laboratory validation approaches are time-consuming, costly, and technically demanding.

Recently, various computational methods have been developed to expedite the identification process of TCR–pMHC interactions by prioritizing the most promising candidates based on TCR and peptide sequence information (Hudson *et al.* 2023). For example, NetTCR (Jurtz *et al.* 2018) encodes TCR and peptide sequences based on the BLOSUM50 matrix (Henikoff and Henikoff 1992). The resulting feature embeddings are concatenated and fed into a convolutional neural network (CNN) (LeCun *et al.* 1998) to predict TCR–pMHC interactions. ERGO (Springer *et al.* 2020) uses an autoencoder and a long short-term memory network (Hochreiter and Schmidhuber 1997) to encode TCR and peptide sequences, respectively, to identify TCR–pMHC interactions. ImRex (Moris *et al.* 2021), a 2D CNN model, was proposed to predict TCR–pMHC interactions based on multi-channel interaction maps. It encodes the feature embeddings of TCR and peptide sequences with physicochemical properties including hydrophobicity, hydrophilicity, mass, and isoelectric point. TITAN (Weber *et al.* 2021) utilizes a 1D CNN with different kernel sizes to encode amino acid-wise information from the BLOSUM62 matrix. Moreover, a bimodal attention network is utilized to assess the significance of each token in the sequences and predict missing links between unseen TCRs and peptides.

The aforementioned methods have achieved great success, yet they still suffer from the shortage of TCR–pMHC interaction data. The majority of the existing methods were designed to accept single-modal input, i.e. sequences or physicochemical features, which limits their abilities to incorporate information from different resources. Moreover, most of these methods were intended to predict solely the TCRβ (the CDR3 region of TCR’s β-chain) and peptides interaction. They cannot handle paired TCR cases where TCRα (the CDR3 region of TCR’s α-chain), TCRβ, and the peptides together are taken into account. To address these issues, we propose a novel multimodal framework dubbed MIX-TPI for predicting TCR–pMHC interactions by mixing the extracted sequence and physicochemical representations. MIX-TPI is applicable to both single-chain TCR and paired TCR scenarios. Particularly, we use CNNs to construct sequence-based extractor (SE) and physicochemical-based extractor (PE), with which the refined sequence and physicochemical features are learned, respectively. These features are then used to form the modality-invariant and modality-specific representations, enabling MIX-TPI to capture the underlying commonalities between different modalities and distinctive characteristics of the specific modality, respectively. Finally, a self-attention fusion layer with different learnable weights is introduced to combine these representations and identify the TCR–pMHC interactions. MIX-TPI is validated on various datasets and evaluation schemes. The experimental results demonstrate the effectiveness and generalization capability of MIX-TPI in comparison with other state-of-the-art methods.

## 2 Materials and methods

### 2.1 Data

In this study, datasets curated from four databases including VDJdb (Bagaev *et al.* 2020), ImmuneCODE (Dines *et al.* 2020), IEDB (Vita *et al.* 2019), and McPAS (Tickotsky *et al.* 2017) are used to evaluate the performance of MIX-TPI. To ensure a fair comparison with other methods, we downloaded two benchmark datasets released in (Weber *et al.* 2021). The first dataset is referred to as VDJdb-TITAN, which is a processed version of the VDJdb database, containing 10 599 known TCR–pMHC interactions of 10 138 TCRs and 87 peptides. The second dataset Immune-TITAN merges the ImmuneCODE database (COVID-19 related database) and the VDJdb-TITAN dataset, resulting in 23 595 known TCR–pMHC interactions of 22 885 TCRs and 192 peptides. According to the maximum sequence length, the fixed padding lengths of TCR *m* and peptide *n* are set to 33 and 20, respectively, in these two datasets. Two different data splitting strategies, i.e. *TCR split* and *strict split* (Weber *et al.* 2021), are used to test the generalization capability of the compared methods. Specifically, *TCR split* specifies the TCRs in the test set being absent in the training set, while *strict split* ensures that neither TCRs nor peptides overlap in test and training sets.

To further validate the generalization ability of MIX-TPI, we collected three mutual exclusive validation datasets from VDJdb and McPAS databases. The first one named VDJdb-ImRex (Moris *et al.* 2021) is curated from the VDJdb database and comprises 14 188 known pairwise associations involving 13 913 TCRs and 117 peptides. VDJdb-ImRex is only used in the training of MIX-TPI. The other two datasets, namely, McPAS-TCRs and McPAS-peptides were created from the McPAS database (Tickotsky *et al.* 2017) to test the performance of MIX-TPI on unseen TCRs and peptides, respectively. McPAS-TCRs comprises 4101 interactions of 4024 TCRs and 46 peptides, excluding all TCRs covered by VDJdb-ImRex. McPAS-peptides includes 736 known associations of 736 TCRs and 10 peptides, excluding all peptides involved in VDJdb-ImRex. The TCRs and peptides in VDJdb-ImRex, McPAS-TCRs, and McPAS-peptides datasets are padded with fixed lengths of 20 and 11, respectively, i.e. m=20 and n=11. Note the three datasets contain merely positive samples of TCR–pMHC interactions and there are no known negative samples, the same number of negative examples are randomly generated for training following the sampling process used in (Moris *et al.* 2021, Weber *et al.* 2021).

The aforementioned datasets, including VDJdb-TITAN, Immune-TITAN, VDJdb-ImRex, McPAS-TCRs, and McPAS-peptides, only contain the sequences of single-chain TCR or more specific TCRβ and peptides. To evaluate the ability of MIX-TPI to handle paired TCR data, we utilize the preprocessed paired TCR dataset provided in (Montemurro *et al.* 2021). The dataset was curated from both the VDJdb and IEDB databases, comprising 2744 known interactions between sequences of 1728 TCRα, 1598 TCRβ, and 17 peptides. We adopted the negative sampling approach described in the original paper, where for each positive interaction, the peptide is fixed, and TCRα and TCRβ were randomly selected from the 10X Genomics (10x Genomics 2019) dataset to form negative samples. The positive-to-negative ratio was configured to 1:5. We refer to this dataset as IEDB-NetTCR, with a maximum padding length of 18 for TCRα and TCRβ, and 9 for peptides (i.e. m=18 and n=9). The information of all datasets used in this study is summarized in Table 1.

**Table 1.** The information of the datasets.

Dataset	#TCRα	#TCRβ	#Peptides	#Interactions
VDJdb-TITAN		10 138	87	10 599
Immune-TITAN		22 885	192	23 595
VDJdb-ImRex		13 913	117	14 188
McPAS-TCRs		4024	46	4101
McPAS-peptides		736	10	736
IEDB-NetTCR	1728	1598	17	2744

### 2.2 Proposed MIX-TPI

**Figure 1.**
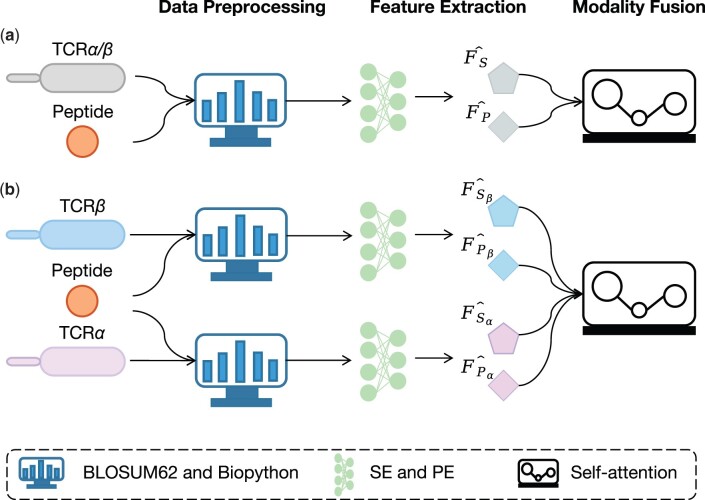
The framework of MIX-TPI to handle both (a) single-chain TCR data, i.e. TCRα/β (α/β-chain) and (b) paired TCR data, i.e. both TCRα and TCRβ. TCRs and peptide sequences are firstly fed into the data preprocessing module, where they are encoded with BLOSUM62 and Biopython (Cock *et al.* 2009) libraries, respectively, to generate the embedding matrices of SE and PE. Subsequently, SE and PE are used to extract sequence and physicochemical features, respectively, with CNNs. Finally, the modality-invariant and modality-specific representations are learned and passed to the self-attention fusion layer to predict the TCR–pMHC interactions.

As shown in Fig. 1, the flowchart of MIX-TPI consists of three stages, i.e. data preprocessing, feature extraction, and modality fusion. The details of these stages are described as follows.

#### 2.2.1 Data preprocessing

To construct the SE embedding, the BLOSUM62 matrix is used to calculate the evolutionary distance of amino acids in TCR sequences and peptide sequences. Specifically, each amino acid is replaced with a log-odds score that corresponds to the substitution pairs of the 20 standard amino acids. Given the maximum padding sequence length of TCR *m* and peptide *n*, we can convert the TCR and peptide sequences into zero-padded embedding matrices of dimensions m×20 and n×20, respectively.

For the PE embedding, we utilize the Biopython library following (Moris *et al.* 2021). The TCR and peptide sequences are fed to calculate four types of physicochemical properties namely hydrophobicity, hydrophilicity, mass, and isoelectric point. These properties provide valuable information about the amino acid sequence and contribute to the overall understanding of protein structure and function. The selection of these properties is also confirmed by their extensive utilization in the field of TCR–pMHC interaction prediction (Ostmeyer *et al.* 2019, Moris *et al.* 2021). For each physicochemical property, we then calculate the pairwise absolute difference between TCR and peptide embeddings to construct an interaction map. Finally, we apply max-min normalization to each interaction map and zero-pad it to the size of m×n to obtain a channel-wise physicochemical embedding of size 4×m×n.

#### 2.2.2 Feature extraction

**Figure 2.**
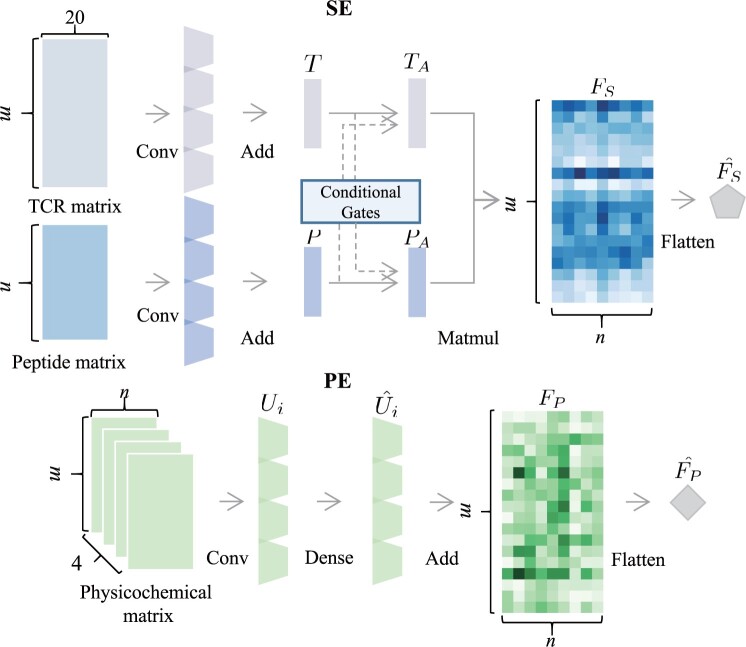
Feature extraction architecture includes sequence-based extractor (upper) and physicochemical-based extractor (lower).

After data preprocessing, two feature extractors, i.e. SE and PE, are constructed to extract refined sequence and physicochemical features, as shown in Fig. 2. The details of SE and PE are described as follows.

### 2.3 Sequence-based extractor

To enhance model generalization and capture local neighboring representations, we utilize 1D CNN with multiple kernel sizes for feature extraction based on the sequence padding matrices. The resulting feature maps are combined through the addition operation to obtain intermediate feature matrices, namely $T\in R^{m\times r}$ for TCR and $P\in R^{n\times r}$ for peptide, where *r* is the predefined number of filters.

Inspired by the advances in cross-modal co-attention mechanisms in visual question answering (Gao *et al.* 2019), we design a cross-modal self-attention module to accurately assess the significance of each modality in relation to the query and key features, taking into account the information from the other modality. Through a feedforward neural network (also known as a dense network), the intermediate feature matrices *T* and *P* are transformed into query/key/value embeddings (Shaw *et al.* 2018), resulting in $T_{Q}/T_{K}/T_{V}\in R^{m\times r}$ and $P_{Q}/P_{K}/P_{V}\in R^{n\times r}$, respectively. These query/key/value embeddings are used to compute the output via a weighted sum of the values, where the weights are derived by the non-linear transformation from the queries and keys. We introduce conditional gates *G* to dynamically regulate the cross-modal information flow with different weights as follows: where the notation ${}_{P\to T}$ denotes the passage of information from peptide to TCR, while ${}_{T\to P}$ indicates the reverse direction. In this equation, σ denotes the *sigmoid* activation function, f(⋅) represents a dense network, and ${\text{Avg}}_{\_}\text{Pool}(\cdot )$ denotes the operation of average pooling (Gao *et al.* 2019).


(1) $$G_{P\to T}=\sigma (f({\text{Avg}}_{\_}\text{Pool}(P)))$$

(2) $$G_{T\to P}=\sigma (f({\text{Avg}}_{\_}\text{Pool}(T)))$$

Then, the query and key embeddings of TCR and peptide are regulated by *G* with the learnable co-attention weights calculated as follows: where ⊙ represents the element-wise multiplication. Accordingly, the TCR attention features $T_{A}\in R^{m\times r}$ and peptide attention features $P_{A}\in R^{n\times r}$ can be calculated with a softmax function (Gibbs 1902) as follows:


(3) $$\hat{T_{Q}}=(1+G_{P\to T})⊙T_{Q}$$

(4) $$\hat{T_{K}}=(1+G_{P\to T})⊙T_{K}$$

(5) $$\hat{P_{Q}}=(1+G_{T\to P})⊙P_{Q}$$

(6) $$\hat{P_{K}}=(1+G_{T\to P})⊙P_{K}$$

(7) $$T_{A}=f(\text{softmax}(\frac{\hat{T_{Q}}{\left(\hat{T_{K}}\right)}^{T}}{\sqrt{r}})\times T_{V})$$

(8) $$P_{A}=f(\text{softmax}(\frac{\hat{P_{Q}}{\left(\hat{P_{K}}\right)}^{T}}{\sqrt{r}})\times P_{V})$$

Afterward, we obtain the fused sequence features as $F_{S}=T_{A}{\left(P_{A}\right)}^{T}\in R^{m\times n}$. $F_{S}$ is flattened into a sequence-based interaction representation vector $\hat{F_{S}}\in R^{mn}$. This vector is subsequently fed into the modality fusion block for further processing. For paired TCR data, $\hat{F_{S_{\alpha }}}\in R^{mn}$ and $\hat{F_{S_{\beta }}}\in R^{mn}$ are used to distinguish between the α and β chains, respectively.

### 2.4 Physicochemical-based extractor

We employ a similar approach to process the channel-wise physicochemical features by using 2D CNN with multiple kernel sizes. This process produces four channel-wise intermediate feature embeddings with the “same” padding as $U_{i}\in R^{m\times n\times r}$, where i∈[1,4]. We then use dense layers to obtain refined feature embeddings $\hat{U_{i}}\in R^{m\times n\times 1}$. The four $\hat{U_{i}}$ embeddings are summed together to generate the feature embeddings $F_{P}\in R^{m\times n}$. Similarly, $F_{P}$ is flattened into a physicochemical-based interaction representation vector $\hat{F_{P}}\in R^{mn}$, which serves as input to the modality fusion block. For paired TCR data, $\hat{F_{P_{\alpha }}}\in R^{mn}$ and $\hat{F_{P_{\beta }}}\in R^{mn}$ represent the α and β chains, respectively.

#### 2.4.1 Modality fusion

**Figure 3.**
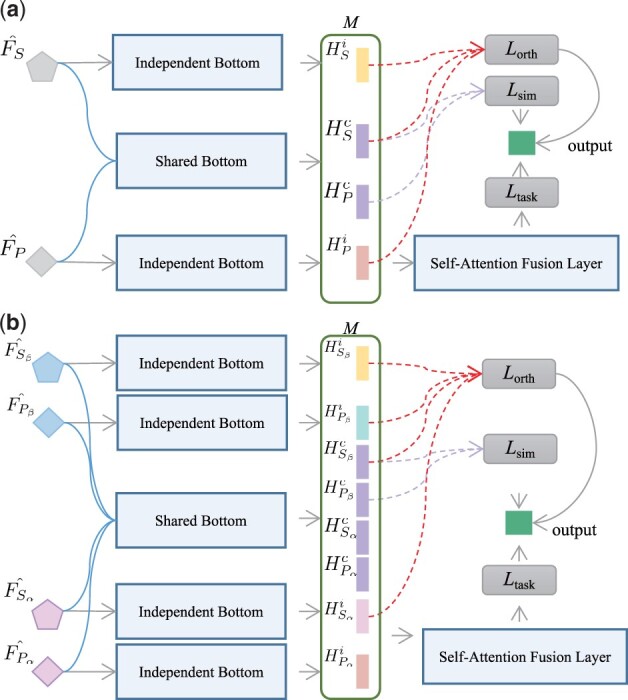
The modality fusion architectures on (a) single-chain TCR datasets and (b) the paired TCR dataset.

For providing a comprehensive view of cross-modal data, we propose a modality fusion block to learn modality-invariant and modality-specific representations. The modality-invariant representation aims to learn the shared representation with distributional similarity constraints (Guo *et al.* 2019) by minimizing the heterogeneity gap. On the other hand, the modality-specific representation focuses on capturing the unique characteristics of each modality. The architecture of the modality fusion block is illustrated in Fig. 3.

The modality fusion block consists of a shared bottom network $f^{c}$ (where *c* refers to a common set of learnable weights) and separate bottom networks $f_{S}^{i}$ and $f_{P}^{i}$ (where *i* refers to individual sets of learnable weights). For single-chain TCR data, we input the calculated $\hat{F_{S}}$ and $\hat{F_{P}}$ to obtain the modality-invariant representations $H_{S}^{c}$ and $H_{P}^{c}$, as well as the modality-specific representations $H_{S}^{i}$ and $H_{P}^{i}$, each with a predefined dimension of *d*. In this work, both the shared and separate bottom networks are implemented with dense layers.

These four representation vectors are concatenated to form a matrix $M=[H_{S}^{c},H_{P}^{c},H_{S}^{i},H_{P}^{i}]\in R^{4\times d}$. To enable each modality to learn latent features from the other modalities, a self-attention fusion layer is applied to *M* (Kiela *et al.* 2019). In this layer, the matrix *M* is transformed into query/key/value embeddings $M_{Q}/M_{K}/M_{V}\in R^{4\times d}$, resulting in a refined matrix $\hat{M}=[\hat{H_{S}^{c}},\hat{H_{P}^{c}},\hat{H_{S}^{i}},\hat{H_{P}^{i}}]\in R^{4\times d}$ as follows:


(9) $$\hat{M}=\text{softmax}(\frac{M_{Q}{\left(M_{K}\right)}^{T}}{\sqrt{d}})M_{V}.$$

Based on $\hat{M}$, we form a concatenated vector $h_{\mathrm{concat}}=[\hat{H_{S}^{c}}\;⊕\;\hat{H_{P}^{c}}\;⊕\;\hat{H_{S}^{i}}\;⊕\;\hat{H_{P}^{i}}]\in R^{4d}$, where ⊕ represents the concatenation operator. We utilize the binary cross-entropy (BCELoss) in conjunction with the AdamW (Loshchilov and Hutter 2019) optimizer to calculate the loss value of the task as follows: where *y* represents the ground-truth labels, and $\hat{y}$ denotes the predicted results through $\hat{y}=\sigma (f(h_{\text{concat}}))$. The total loss of MIX-TPI is defined as $L=L_{\text{task}}+\lambda L_{\text{repre}}$, where λ is the tuning parameter, and the composite loss function $L_{\text{repre}}$ combines $L_{\text{sim}}$ and $L_{\text{orth}}$ to regulate the learning of modality-invariant and modality-specific representations, respectively. Especially, $L_{\text{sim}}$ aims to minimize the gaps between the modality-invariant representations in the shared bottom network $f^{c}$, thereby aligning these representations into the shared vector space (Hazarika *et al.* 2020). To achieve this, we adopt the central moment discrepancy (CMD) method (Zellinger *et al.* 2017) to calculate $L_{\text{sim}}$. The CMD distance metric measures the dissimilarity between the distributions of two representations by evaluating their discrepancies in order-wise moments. As the two distributions become more similar, the CMD distance decreases accordingly. The calculation of $L_{\text{sim}}$ is as follows: where *K* denotes the boundary of central moments and is usually set to 5. The empirical expectation vector of a sample *X* is reached as $E(X)=\frac{1}{\left|X\right|}\sum_{x\in X}^{}$. The vector $C_{K}(X)=E({\left(x-E(X)\right)}^{k})$ represents the collection of all *k*-th order sample central moments in *X*. The orthogonal loss $L_{\text{orth}}$ is proposed to facilitate the learning of non-redundant features in both modality-invariant and modality-specific representations. It ensures that these representations are constrained to orthogonal subspaces. Hence, $L_{\text{orth}}$ is calculated as follows:


(10) $$L_{\text{task}}=-y\;\text{log}(\hat{y})-(1-y)\;\text{log}(1-\hat{y})$$

(11) $$L_{\text{sim}}={\text{CMD}}_{K}(H_{S}^{c},H_{P}^{c})$$

(12) $${\text{CMD}}_{K}(X,Y)={\left\|E(X)-E(Y))\right\|}_{2}+\sum_{k=2}^{K}{\left\|C_{K}(X)-C_{K}(Y))\right\|}_{2}$$

(13) $$L_{\text{orth}}=\sum_{t\in \{S,P\}}{\left\|H_{t}^{c^{T}}H_{t}^{i}\right\|}_{F}^{2}+{\left\|H_{S}^{i^{T}}H_{P}^{i}\right\|}_{F}^{2}$$

Similarly, for paired TCR data, the representation matrices $\left\{\hat{F_{S_{\alpha }}},\hat{F_{P_{\alpha }}},\hat{F_{S_{\beta }}},\hat{F_{P_{\beta }}}\right\}$ are fed to $f^{c}$ and $f^{i}$ to obtain corresponding modality-invariant representations $\left\{H_{S_{\alpha }}^{c},H_{P_{\alpha }}^{c},H_{S_{\beta }}^{c},H_{P_{\beta }}^{c}\right\}$ and modality-specific representations $\left\{H_{S_{\alpha }}^{i},H_{P_{\alpha }}^{i},H_{S_{\beta }}^{i},H_{P_{\beta }}^{i}\right\}$. Accordingly, $L_{\text{sim}}$ and $L_{\text{orth}}$ can be rewritten as follows: where $\phi =\{(S_{\alpha },P_{\alpha }),(S_{\alpha },S_{\beta }),(S_{\alpha },P_{\beta }),(P_{\alpha },S_{\beta })(P_{\alpha },P_{\beta }),(S_{\beta },P_{\beta })\}$.


(14) $$L_{\text{sim}}=\frac{1}{6}\sum_{t_{1},t_{2}\in \phi }{\text{CMD}}_{K}(H_{t_{1}}^{c},H_{t_{2}}^{c})$$

(15) $$\begin{array}{c} L_{\text{orth}}=\sum_{t\in \{S_{\alpha },P_{\alpha },S_{\beta },P_{\beta }\}}{\left\|H_{t}^{c^{T}}H_{t}^{i}\right\|}_{F}^{2}+\sum_{t_{1},t_{2}\in \phi }{\left\|H_{t_{1}}^{i^{T}}H_{t_{2}}^{i}\right\|}_{F}^{2} \end{array}$$

## 3 Results

### 3.1 Experiment setup

To evaluate the performance of different data splitting strategies, this study adopts cross-validation based on two primary evaluation metrics, i.e. the area under the receiver-operating characteristic curve (AUC) and the area under the precision-recall curve (AUPR). AUC is widely used in this field (Springer *et al.* 2020, Moris *et al.* 2021, Weber *et al.* 2021), while AUPR is used to assess the performance on the unbalanced dataset (i.e. IEDB-NetTCR). The implementation of MIX-TPI is carried out in Python and PyTorch v1.10.0, and the default parameter settings are presented in Table 2.

**Table 2.** Parameter settings.

No. of filters (*r*)	[64, 256]	Feature size (*d*)	[64, 256]
Weight of $L_{\text{repre}}$ (λ)	[0.1, 0.3]	CNN kernel sizes	{3, 5, 9, 11}
Epoch	50	Batch size	128
Dropout rate	[0.3, 0.5]	Learning rate	[1e−5, 1e−3]

### 3.2 Comparison with state-of-the-art methods

For performance evaluation, MIX-TPI is pitted against four state-of-the-art methods, namely NetTCR (Jurtz *et al.* 2018), ERGO (Springer *et al.* 2020), ImRex (Moris *et al.* 2021), and TITAN (Weber *et al.* 2021). The evaluation is conducted on the VDJdb-TITAN and Immune-TITAN datasets. For the sake of fairness, all the compared methods are provided with the same inputs as MIX-TPI and configured with their default parameter settings as described in their respective articles.

**Figure 4.**
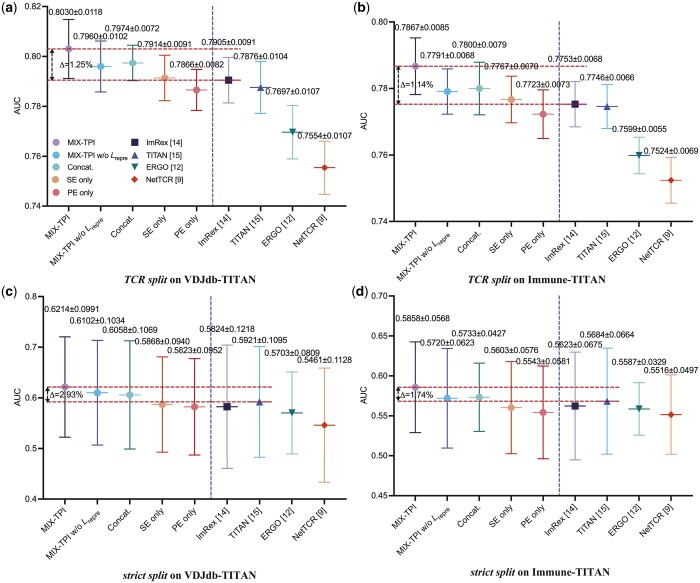
The average AUC of the compared methods on the VDJdb-TITAN and Immune-TITAN datasets by different data splitting strategies. The left side of each subfigure represents the results of MIX-TPI’s variants, while the right side of each subfigure denotes the results of the compared state-of-the-art methods.

The right side of each subfigure in Fig. 4 presents the results of the compared methods evaluated with 10-fold cross-validation. MIX-TPI demonstrates superior performance with the highest average AUC values compared with the other methods. Specifically, it outperforms the runner-ups by 1.25%, 1.14%, 2.93%, and 1.74% on the following data splitting strategies: *TCR split* on VDJdb-TITAN, *TCR split* on Immune-TITAN, *strict split* on VDJdb-TITAN, and *strict split* on Immune-TITAN, respectively. We also observe that all the compared methods perform worse on *strict split* than on *TCR split*, indicating the heterogeneity of peptides and the difficulty in generalizing to the associations involving completely unseen TCRs and peptides. Among these methods, TITAN and ImRex perform relatively better, because they utilize either sequence-based features or physicochemical-based features, but not both. In contrast, MIX-TPI reaches superior generalization capability and robust prediction performance, primarily due to its incorporation of both sequence-based and physicochemical-based features, along with the comprehensive view of subspace representation learning.

### 3.3 Ablation study

The self-attention fusion layer, representation loss $L_{\text{repre}}$, SE, and PE are the important components accounting for the performance improvement reached by MIX-TPI. We conduct an additional experiment to evaluate their effectiveness. In the experiment, we replace the self-attention fusion layer with a simple concatenation operation and compare the performance of the modified model with the original MIX-TPI. The output embeddings $\hat{F_{S}}$ and $\hat{F_{P}}$ of SE and PE are respectively fed into separate feedforward neural networks for predicting the TCR–pMHC interactions. The results are shown on the left side of each subfigure in Fig. 4. The comparison reveals that the AUC of MIX-TPI degrades by about 1% without the self-attention fusion layer (denoted as Concat.) and the representation loss (denoted as MIX-TPI w/o $L_{\text{repre}}$). This finding suggests that the self-attention fusion layer and learning modality-invariant and modality-specific representations play a crucial role in enhancing the performance of MIX-TPI.

Moreover, the results demonstrate that the single-modal variants (SE only and PE only) show a decrease in AUC compared with the combined MIX-TPI model. Specifically, the AUC decreases by 1.16%/1.34%, 1.00%/1.44%, 3.46%/3.91%, and 2.55%/3.15% on *TCR split* on VDJdb-TITAN, *TCR split* on Immune-TITAN, *strict split* on VDJdb-TITAN, and *strict split* on Immune-TITAN, respectively. This suggests that the combination of sequence and physicochemical features in MIX-TPI provides effective supplementary information for prediction. Importantly, despite the degradation in performance, all variants of MIX-TPI still achieve superior performance compared with other state-of-the-art methods, demonstrating the robustness of the proposed MIX-TPI model.

### 3.4 Evaluation on mutual exclusive evaluation datasets

In order to evaluate the generalization capability of the models in a real-world scenario, mutual exclusive evaluation is conducted using VDJdb-ImRex and McPAS datasets for training and testing, respectively. Two subsets of McPAS are utilized for testing: one excluded TCRs appearing in VDJdb-ImRex (McPAS-TCRs test set), and the other removed peptides contained in VDJdb-ImRex (McPAS-peptides test set). The results of 10-fold CV on VDJdb-ImRex and mutual exclusive evaluation set testing on McPAS are summarized in Table 3, where MIX-TPI achieves the highest average AUC in all cases. Comparison of the results between Table 3(a) and (b) reveals that all the compared methods fail to maintain their generalization capability in mutual exclusive evaluation set testing, leading to a significant decline in performance when compared with the results of cross-validation. This highlights the technical challenge of evaluating the generalization capability of models for real-world applications through mutual exclusive evaluation set testing.

**Table 3.** Average AUC comparison of the 10-fold CV on VDJdb-ImRex and mutual exclusive evaluation on McPAS.

Methods	(a) 10-Fold CV on VDJdb-Im-Rex	(b) Mutual exclusive evaluation on McPAS	
		McPAS-TCRs	McPAS-peptides
NetTCR (Jurtz *et al.* 2018)	0.6366±0.0127	0.5752±0.0183	0.5088±0.0357
ERGO (Springer *et al.* 2020)	0.6444±0.0116	0.5820±0.0122	0.5230±0.0260
ImRex (Moris *et al*. 2021)	0.6645±0.0110	0.6012±0.0072	0.5317±0.0286
TITAN (Weber *et al*. 2021)	0.6624±0.0095	0.5980±0.0171	0.5298±0.0246
MIX-TPI w/o $L_{\text{repre}}$	0.6806±0.0073	0.6116±0.0126	0.5344±0.0271
MIX-TPI	**0.6890** ± **0.0104**	**0.6182** ± **0.0099**	**0.5462** ± **0.0235**

The boldface values indicate the best performance.

### 3.5 Evaluation on the paired TCR dataset

We also conduct a thorough performance analysis of MIX-TPI in processing paired TCR data, peptide-specific AUC comparison, the impact of the representation loss $L_{\mathrm{repre}}$ on the vector subspace of representations, and the decision-making process of self-attention in the fusion stage. We utilize the IEDB-NetTCR dataset for our experiments and adopt 5-fold CV as per the settings described in NetTCR2.0 (Montemurro *et al.* 2021), only which is capable of handling paired TCR data among the compared methods.

#### 3.5.1 Handling paired TCR data

Since MIX-TPI is compatible with handling paired TCR data, we use NetTCR2.0 as a benchmark for performance comparison. NetTCR2.0 employs 1D CNNs to encode the sequences of peptides, TCRα, and TCRβ and then concatenate the encoded representations to predict the paired TCRαβ-pMHC interactions. As shown in Table 4, MIX-TPI achieves a higher AUC (by 1.38%) and AUPR (by 2.37%) than NetTCR2.0. This result demonstrates the robustness of MIX-TPI in handling the unbalanced nature of the IEDB-NetTCR dataset. The simple concatenation feature fusion in NetTCR2.0 may limit its performance in effectively capturing the TCR–pMHC interaction patterns. Interestingly, even without TCRα (denoted as MIX-TPI w/o α) or TCRβ (denoted as MIX-TPI w/o β), MIX-TPI still outperforms NetTCR2.0 with paired TCR data. This may attribute to the fact that NetTCR2.0 only considers sequence-based features. In contrast, MIX-TPI introduces physicochemical-based features in addition to sequence-based features, depicting the TCR–pMHC interactions from different perspectives.

**Table 4.** Average AUC and AUPR comparison on IEDB-NetTCR.

Methods	AUC	AUPR
NetTCR2.0	0.8963±0.0080	0.8177±0.0089
MIX-TPI w/o α	0.9034±0.0071	0.8336±0.0090
MIX-TPI w/o β	0.9028±0.0048	0.8325±0.0063
MIX-TPI w/o $L_{\text{repre}}$	0.9046±0.0058	0.8339±0.0077
MIX-TPI	**0.9101** ± **0.0064**	**0.8415** ± **0.0086**

The boldface values indicate the best performance.

#### 3.5.2 Peptide-specific AUC comparison

**Figure 5.**
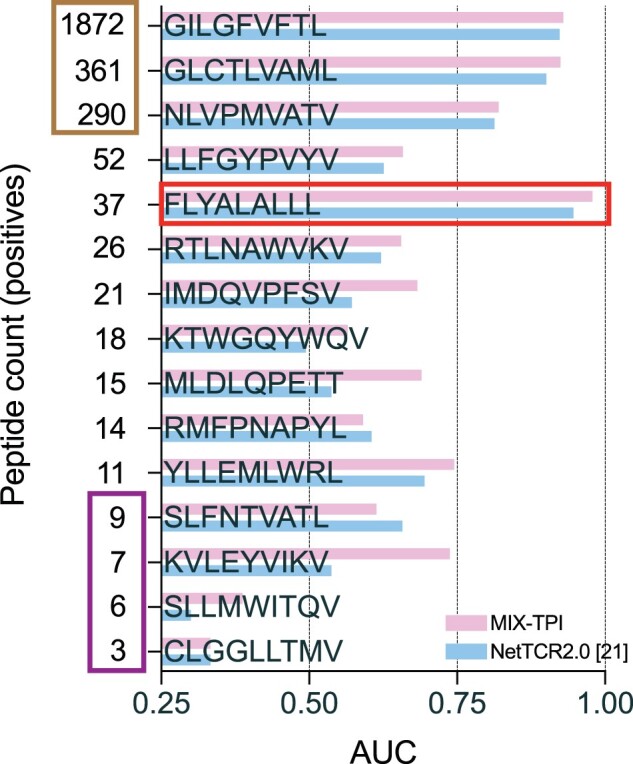
Comparison of MIX-TPI and NetTCR2.0 in terms of peptide-specific average AUC on peptides with at least three positive examples.

We conducted further analysis on the impact of the number of interactions per peptide on the performance. Figure 5 shows the peptide-specific average AUC of MIX-TPI and NetTCR2.0 using 5-fold CV. MIX-TPI consistently outperforms NetTCR2.0 in most peptides. Peptides with over 200 positive interactions have an average AUC of 0.89, while peptides with <10 positive interactions have an average AUC of 0.52. Furthermore, we observe a decrease in AUC as the number of peptide interactions decreases, except for peptide FLYALALLL which has an AUC of 0.97. This outlier can be explained by the high dissimilarity between its positive and negative TCRs, as reported in NetTCR2.0 (Montemurro *et al.* 2021).

#### 3.5.3 Analysis of vector subspace representations

**Figure 6.**
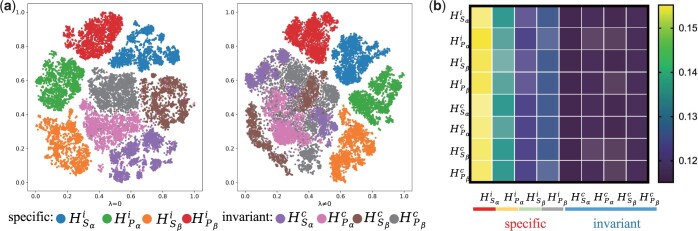
Representation embedding analysis. (a) t-SNE (Van der Maaten and Hinton 2008) visualization of vector subspace of modality-invariant and modality-specific representations. (b) Average self-attention scores. The rows denote queries and the columns denote keys. Transparency is used to further differentiate the scores of each column, with higher transparency indicating lower scores.

The ability of MIX-TPI to learn the vector subspace representations is also investigated in this section. Figure 6a displays the vector subspaces of modal-invariant representations $\{H_{S_{\alpha }}^{c}$, $H_{P_{\alpha }}^{c}$, $H_{S_{\beta }}^{c}$, $H_{P_{\beta }}^{c}\}$ and modal-specific representations $\{H_{S_{\alpha }}^{i}$, $H_{P_{\alpha }}^{i}$, $H_{S_{\beta }}^{i}$, $H_{P_{\beta }}^{i}\}$ for samples in the test set. The results show that when the representation loss $L_{\text{repre}}$ is not included (i.e. λ=0), the modality-invariant representations cannot be learned. However, when $L_{\text{repre}}$ is involved (i.e. λ≠0), there is a clear fusion effect observed among the modality-invariant representations. It is worth noting that the modal-specific representations remain scattered, as they are supposed to be distinct for each modality. However, their distributions are more condensed when $L_{\mathrm{repre}}$ is utilized. The results demonstrate that $L_{\text{repre}}$ plays a crucial role in learning the modality-invariant representations and improving the performance of MIX-TPI.

#### 3.5.4 Importance of the learned representations through attention visualization

We investigate the importance of the learned representations by visualizing the average attention distribution in the fusion stage over the test set. As shown in Fig. 6b, each row represents the probability distribution of each representation embedding, averaged over all test samples, while each column shows the significance of a specific representation $H_{\left\{S_{\alpha }/P_{\alpha }/S_{\beta }/P_{\beta }\right\}}^{\left\{c/i\right\}}$ to all enhanced representations ${\hat{H}}_{\left\{S_{\alpha }/P_{\alpha }/S_{\beta }/P_{\beta }\right\}}^{\left\{c/i\right\}}$.

It is observed that the four modality-invariant representations have similar patterns, which can be attributed to their shared vector subspaces. On the other hand, the contributions of the four modality-specific representations are distinctive because of their orthogonality constraints. Although the modality-specific representations seem to have higher importance than the modality-invariant representations, these representations provide varying degrees of information to the results.

## 4 Conclusion

We proposed a multimodal computational framework named MIX-TPI for the prediction of TCR–pMHC interactions. The sequence-based extractor and physicochemical-based extractor were shown to effectively capture feature embeddings. The modality fusion stage of MIX-TPI incorporates similarity and orthogonality constraints to facilitate the learning of modality-invariant and modality-specific representations. These constraints allow the model to capture both commonalities and diversities across different modalities, effectively modeling the intricate relationships between them. The self-attention fusion layer was then employed to fuse these representations to predict TCR–pMHC interactions. The effectiveness and reliability of MIX-TPI were demonstrated on VDJdb-TITAN and Immune-TITAN datasets using two data splitting strategies. The generalization capability of MIX-TPI for real-world applications was further validated through mutual exclusive evaluation on McPAS datasets. Experiments conducted on the paired TCR dataset, specifically IEDB-NetTCR, validated the capability of MIX-TPI in processing multimodal data with high flexibility. The results also demonstrated its proficiency in achieving desired vector subspace learning, as well as showcasing the self-attention decision process in the fusion stage.

While MIX-TPI has demonstrated promising performance, there are still areas where further improvements can be made. The accuracy of negative samples for TCR–pMHC interactions is crucial. In this study, negative samples were generated through shuffling or sampling from 10X Genomics data. However, these negative samples may contain false negatives. To address this issue, positive-unlabeled learning approaches (Zeng *et al.* 2020, Jiang *et al.* 2023) could be utilized to select reliable negative TCR–pMHC interactions. Furthermore, MIX-TPI only incorporates data from the CDR3 regions of the TCR. Integration of other valuable information, such as CDR1, CDR2, V/D/J gene usage, and other physicochemical features (Lanzarotti *et al.* 2019, Wang and Zou 2023), can provide a more comprehensive understanding of the interaction patterns between TCR and pMHC from different perspectives.

## Data Availability

The source code of MIX-TPI and the test data are available at: https://github.com/Wolverinerine/MIX-TPI.
